# Determination of Nanoparticles and Elements in Blue Mussels (*Mytilus edulis*) along the Norwegian Coastline

**DOI:** 10.1021/acs.jafc.4c04721

**Published:** 2024-11-07

**Authors:** Are Sæle Bruvold, Stig Valdersnes, André Marcel Bienfait, Monica Sanden, Katrin Loeschner

**Affiliations:** a Institute of Marine Research (IMR), PO Box 1870 Nordnes, Bergen N-5817, Norway; b Department of Chemistry, University of Bergen, PO Box 7803, Bergen N-5020, Norway; c National Food Institute, Technical University of Denmark, Kemitorvet 201, Kgs Lyngby DK-2800, Denmark

**Keywords:** nanoparticles, SP-ICP-MS, seafood, mussels, survey, elements

## Abstract

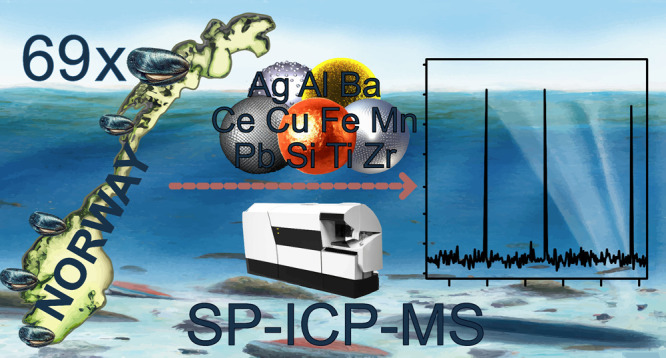

Our work aimed to examine nanoparticle levels in 69 distinct pooled mussel samples along the Norwegian coastline, considering samples from different environmental contexts, including natural locations, potentially polluted hotspots, and mussel farms. Single-particle ICP-MS was utilized to determine particle mass and number concentrations at environmentally relevant levels in addition to the total content of 11 elements: aluminum, barium, cerium, copper, iron, manganese, lead, silicon, silver, titanium, and zirconium. Results showed nanoparticle mass concentrations of few ng/g up to tens of μg/g and number concentrations of 10^6^ to 10^9^ particles/g (wet weight). Certain urban and industrially impacted locations were linked to increased levels of, e.g., silver, lead, cerium, zirconium, and titanium NPs. Farmed mussels exhibited lower concentrations. However, natural variations were considerable, and impacted locations mostly did not differ from the highest levels in pristine areas. The study presents the first extensive survey of NPs of 11 different elements in marine biota and provides evidence of increased levels of NPs in areas with anthropogenic activities.

## Introduction

Blue mussels (*Mytilus edulis*) efficiently bioaccumulate pollutants by pumping and filtering large volumes of water over their ciliated gills. Blue mussels are well suited as bioindicators because they are sedentary, are tolerant to pollutants, have a long life span, and have wide geographical distribution and their physiology has been extensively studied.^1^ They are commonly used for coastal pollution monitoring.^2−4^ They are also the most studied organisms in terms of the ecotoxicology of nanoparticles (NPs).^5^ However, relatively little is known about the concentrations and geographical distributions of NPs in mussels. Singl- particle inductively coupled plasma mass spectrometry (SP-ICP-MS) allows the determination of particle masses, mass-equivalent spherical diameters, and concentrations at low, environmentally relevant levels. NPs are commonly defined as exhibiting an external dimension in the range of 1 to 100 nm,^6^ although some define the nanoscale to encompass 1 to 1000 nm.^7^ SP-ICP-MS determines elemental mass, and thus, the resulting sizes, generally mass-equivalent spherical diameters, are based on assumptions regarding composition. Because the technique is not selective to the specific size range of NPs, detections within the method’s working range (defined operationally by the measurement procedure,^8,9^ e.g., by the instrumentation and by the sample preparation and data processing, and typically between a few tens and hundreds of nm) are reported as NPs. Existing studies have investigated bivalves and seafood for the presence of selected NPs such as silver (Ag),^10−13^ zinc (Zn) oxide,^14^ and titanium (Ti) dioxide NPs.^11,15−17^ These studies typically found particle mass concentrations in the range of nanogram per gram to tens of μg/g and particle number concentrations of 10^6^ to 10^11^ particles/g (dry or wet weight). NP types other than Ag, Zn oxide, and Ti oxide were also detected in shellfish including mercury (Hg)-containing NPs;^18^ yttrium (Y)-, lanthanum (La)-, cerium (Ce)-, praseodymium (Pr)-, gadolinium (Ga)-, and neodymium (Nd)-containing NPs;^19^ aluminum- (Al), iron- (Fe), and silicon (Si)-containing NPs;^20^ and copper (Cu)-containing NPs.^21^ However, the trueness of the determined NP sizes and concentrations cannot be assessed in the absence of in-matrix reference materials. Due to the lack of method intercalibration, standardization, and transparency, comparing operationally defined SP-ICP-MS data across studies is challenging. For this reason, data on geographical distributions and potential impacts of human activities on the accumulation of NPs in mussels are lacking.

Along the Norwegian coast, a variety of anthropogenic sources may release NPs to the marine environment, which may be taken up by mussels. The three largest cities, Oslo, Bergen, and Trondheim, are all situated on the coast and hotspots for incidental NPs from, e.g., road dust^22,23^ as well as engineered NPs released through, e.g., wastewater or from painted surfaces.^24,25^ Tyssedal is a heavily industrially impacted site situated in a fjord with, e.g., current or historic melting plants for Al, Zn, and TiO_2_. The submarine tailing deposits present in Ranfjorden (current) and in Repparfjorden (historic) have yet to undergo investigation for NP content and could contain a substantial quantity of incidental NPs. Simultaneously, mussel farming is widespread along the coast, with the annual production of farmed mussels reaching 2612 tons in Norway in 2022.^26^ The Norwegian Food Safety Authority establishes and oversees production areas for bivalves to ensure food safety, in compliance with EC 2017/625.

In addition to requirements on microbiological quality, maximum levels (MLs) are set for certain contaminants in feed and food including bivalve mollusks.^27,28^ Environmental quality standards (EQSs) have also been proposed for some contaminants, including multiple trace metals.^29^ However, MLs for NPs do not exist, and the aforementioned challenges with comparing data across studies make the risk assessment and future establishment of EQSs and MLs for NPs difficult. We recently developed and validated a cost-efficient method based on a simple protease mixture for sample preparation and a novel open-source data processing using gold NPs, which can be used for determining NPs of different elements in biota including mussel matrix.^30^ This signal processing employed maximum-peak-intensity-based signal discrimination based on Poisson statistics. Peaks exceeding an intensity threshold were integrated from a rolling median baseline as an approximation of the background noise.

The objective of this study was to investigate the levels of NPs in blue mussels across the Norwegian coastline. Wild mussels from anticipated pristine locations (“natural”), wild mussels from potentially polluted hotspots (“anthropogenic”), and farmed blue mussels (“farmed”) were analyzed for NPs using SP-ICP-MS. The data were used to examine the environmental variations in NP concentrations and the levels in mussels used for food and to assess the anthropogenic impact from hotspot sources such as cities and mining waste disposal. The NPs’ concentrations were compared with the total element content, and the contributions of the seasonality and mussel length (as a proxy for age) to the observed variations were examined.

### Sampling and Sample Treatment

**Figure 1:**
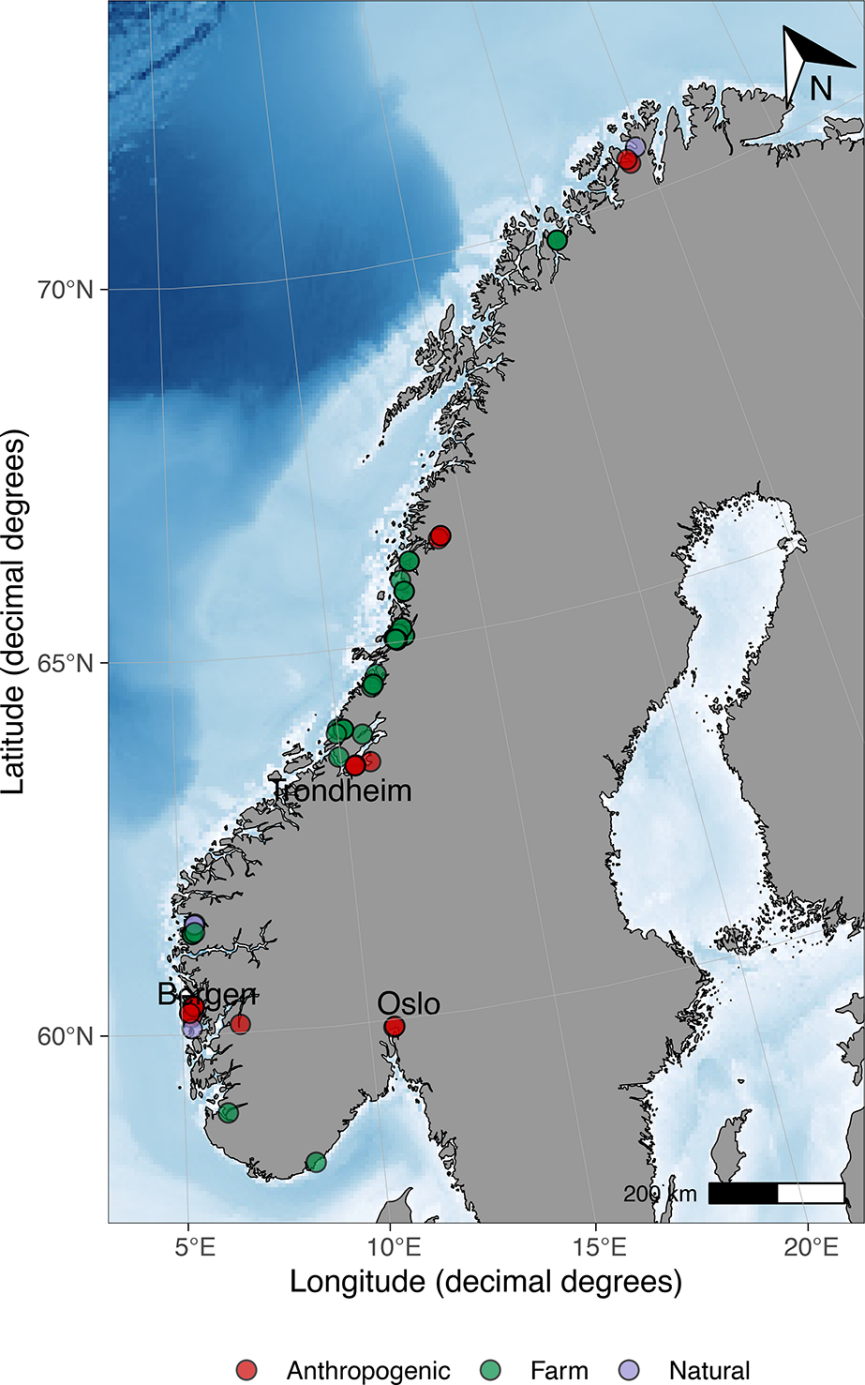
Sample locations across the Norwegian coast, with locations classified as mussel farms (green), natural (purple), or anthropogenic (red) based on known anthropogenic sources such as vicinity to major cities (Bergen, Oslo, and Trondheim), mining waste disposal (Repparfjorden and Ranfjorden), or industrial pollution (Tyssedal). Created using ggOceanMaps^31^ and NOAA ETOPO 2022 15 Arc-Second Global Relief Model.^32^

Samples of blue mussels (*Mytilus edulis*) were acquired through the national monitor program for bivalves,^2^ surveys performed by the Institute of Marine Research (IMR) in Bergen, Norway, or collected manually. An overview of all sample locations is presented in Figure 1. Coordinates, shell lengths, and sampling dates can be found in Table S1. Locations were distributed along the Norwegian coastline and classified as mussel farms (green), natural (purple), or anthropogenic (red) based on known anthropogenic sources such as major cities (Bergen, Oslo, and Trondheim), mining waste disposal (Ranfjorden and Repparfjorden), or industrial pollution (Tyssedal). Forty-seven locations were mussel farms, 18 were classified as potentially impacted by human activity, and 4 were assumed to be pristine. Sampling collected at least 10 samples per location with sizes of approximately 3–5 cm where available and at varying depths above 10 m. The collected samples were frozen at −20 °C within a day.

Aggregate mussel samples were prepared by extracting the mussel tissue based on an internal standardized procedure that is described briefly in the following text. The mussels were allowed to thaw overnight before they were opened by cutting the sphincter muscle. Visible foreign bodies were removed manually or by rinsing with ultrapure water (UPW). Mussels were placed upright to dry for approximately 5 min. Thereafter, the tissue was extracted with a blunt knife and was briefly washed by mixing into 0.5 L ultrapure water (UPW). The suspension containing UPW and mussel tissue was then transferred to a sieve for 5 min to drain the UPW, and the tissue was transferred to a plastic container and then frozen until homogenization. During this process, physiological measurements of the weight and length of the mussels were taken. Homogenization was performed using a benchtop homogenizer (Polytron PT-2100, Kinematica AG, Switzerland) for approximately 2 min. The homogenizer was rinsed in three subsequent UPW baths for each sample, which were renewed for six samples. The homogenized samples were frozen until sample preparation. For total element analysis, the homogenates were additionally freeze-dried by using a Labconco Freezone model 775030. The ratios between dry and wet weight were acquired to be able to back-calculate the wet weight equivalent.

### Reagents

Ionic standards of Ag (S-Ag-1.125, SPS, Singapore), gold (SS-1118N, Spectrascan, Ski, Norway), barium (Ba) (SS-1216, Spectrascan, Ski, Norway), Ce (16734-100 ML, Sigma-Aldrich), Cu (58921-100 ML-F, Sigma-Aldrich), Mn (SS-1105, Spectrascan, Ski, Norway), Pb (SS-1117, Spectrascan, Ski, Norway), Si (08729-100 ML, Sigma-Aldrich), Ti (SS-1164, Spectrascan, Ski, Norway), and a multielement standard containing Fe, Al, Mn, Pb, Cu, Ba, and Ag (SS-6083S, Spectrascan, Ski, Norway) were used for calibration. Spherical gold NPs of 60 nm (50 mg/L NanoXact, lot TJC0086, nanoComposix, San Diego, CA, USA) were employed to determine the transport efficiency. The protease from *Bacillius* sp. (Protamex, Merck) used for digestion was purchased from Sigma-Aldrich, Saint Louis, MO. All aqueous dilution and rinsing were performed with UPW with resistivity of 18.2 MΩ·cm at 25 °C (Elix Progard TNP and Milli-Q Advantage A10, Merck Millipore, MA, USA). Nitric acid (65% EMSURE for analysis, Merck) was used for the ionic standards and for ICP-MS rinsing. Laboratory grade Triton X-100 (Sigma-Aldrich) and hydrochloric acid (TraceSelect, Fluka Analytical, Switzerland) were also used for rinsing.

### Sample Preparation

The homogenized mussel samples were allowed to thaw overnight and stored in a refrigerator prior to analysis. For the SP-ICP-MS analysis, 1 ± 0.02 g of blue mussel tissue was weighed into polypropylene tubes in triplicates. For the procedural blanks (seven per analysis day), 1 mL of UPW treated with the benchtop homogenizer to mimic sample homogenization was added instead. A volume of 0.6 mL of a 200 g/L Protamex stock solution was added to the samples followed by 2.5 mL of UPW prior to mixing with a vortex shaker (MS1 minishaker, IKA, Germany), ensuring that no tissue was adhering to the walls. Samples were incubated at 50 °C overnight on a customized angled sample holder at 280 rotations per minute using an incubator heat shaker (Heidolph Unimax, Schwabach, Germany). On the next day, samples were diluted with UPW to reach final concentrations corresponding to 1 g of mussel tissue per liter. Vortex shaking was performed after each dilution step.

The total element analysis was based on a previously described method.^33^ Approximately 0.2 g of freeze-dried tissue was weighed into UltraWAVE PTFE sample tubes (Milestone, Italy) followed by 1 mL of UPW and 2 mL of concentrated nitric acid. UPW was treated in parallel and as procedural blanks. The samples were then fully digested using an UltraWAVE Microwave Digestion System (Milestone, Italy). After the samples were allowed to cool to ambient temperature, they were transferred to polypropylene tubes and gravimetrically diluted to 25 g (corresponding to 25 mL) using UPW.

### Data Acquisition and Processing

For SP-ICP-MS analysis, an Agilent 8900 Triple Quadrupole ICP-MS instrument (Agilent Technologies, Santa Clara, CA, USA) with reaction and collision gas capability and an SPS 4 autosampler was used for sequential analysis of the 11 different elements. It was equipped with a concentric MicroMist nebulizer in borosilicate glass and a Scott-type double pass spray chamber in quartz (temperature 2 °C). Hydrogen reaction gas was used to alleviate spectral interference on Fe at *m*/*z* 56 and Si at *m*/*z* 28. Interference on Ti at *m*/*z* 48 and TiO^+^ at *m*/*z* 64 was removed using hydrogen and oxygen combined with a mass shift. Additional parameters for each measured isotope are given in Table S2. A three-point calibration curve was fitted, with standards measured at the start and end of each day at concentrations of 0, 20, and 200 μg/L. Gold NP was freshly prepared in UPW to a concentration of 1250 ng/L and used to calculate the response factor, transport efficiency, and mass flow rate of the sample being delivered to the detection system. Signal intensities for each element were recorded using a dwell time of 100 μs with an acquisition time of 45 s. A three-step rinsing procedure was employed between each sample using a 1% mixture of Triton-X, HCl, and HNO_3_ followed by 5% HNO_3_ and finally UPW, each at 0.5 RPS for 30 s. Instrument tuning was assessed, and the uptake rate was measured gravimetrically over at least 20 min before the start of the instrumental analysis. Ionic and NP standards were analyzed at the beginning and end of each analytical sequence. One specific mussel homogenate sample was analyzed in duplicate on each analysis day as a quality control for determination of repeatability and intermediate precision. The instrument raw data were processed using a previously described algorithm-based particle discrimination using max peak intensity as detailed previously.^30^ Blank subtraction was performed using the mean of procedural blanks containing enzyme and homogenized UPW. The size or mass per particle detection limit was set to the highest value across all days to ensure similar false negative rates (Table S3) defined by a false positive rate of one particle per minute, assuming Poisson distributed noise. Detection and quantification limits in terms of particle mass and particle number concentrations were determined for each day from the procedural blanks, and the mean value across all days was used as the detection limit for all days.

For the total element analysis, the same instrument was used as that for the single-particle analysis (Table S2). The detection limit was set as 3× the standard deviation of 12 procedural blanks, except for silver, where six blanks were utilized. These were also utilized for blank subtraction. The dry weight percentage was employed as a multiplicative factor to back-calculate the corresponding wet weight for the total elements. Concentrations below the detection limit were excluded from visualizations, cluster analysis, and the calculation of particle fractions. Cluster analysis using agglomerative hierarchical clustering was performed using the hclust package in R version 4.2.1. Clusters were calculated using Ward’s minimal increase of sum-of-squares method using Euclidean distances following standardization via *z*-score.

## Results and Discussion

### Distributions and Patterns of NPs

**Figure 2:**
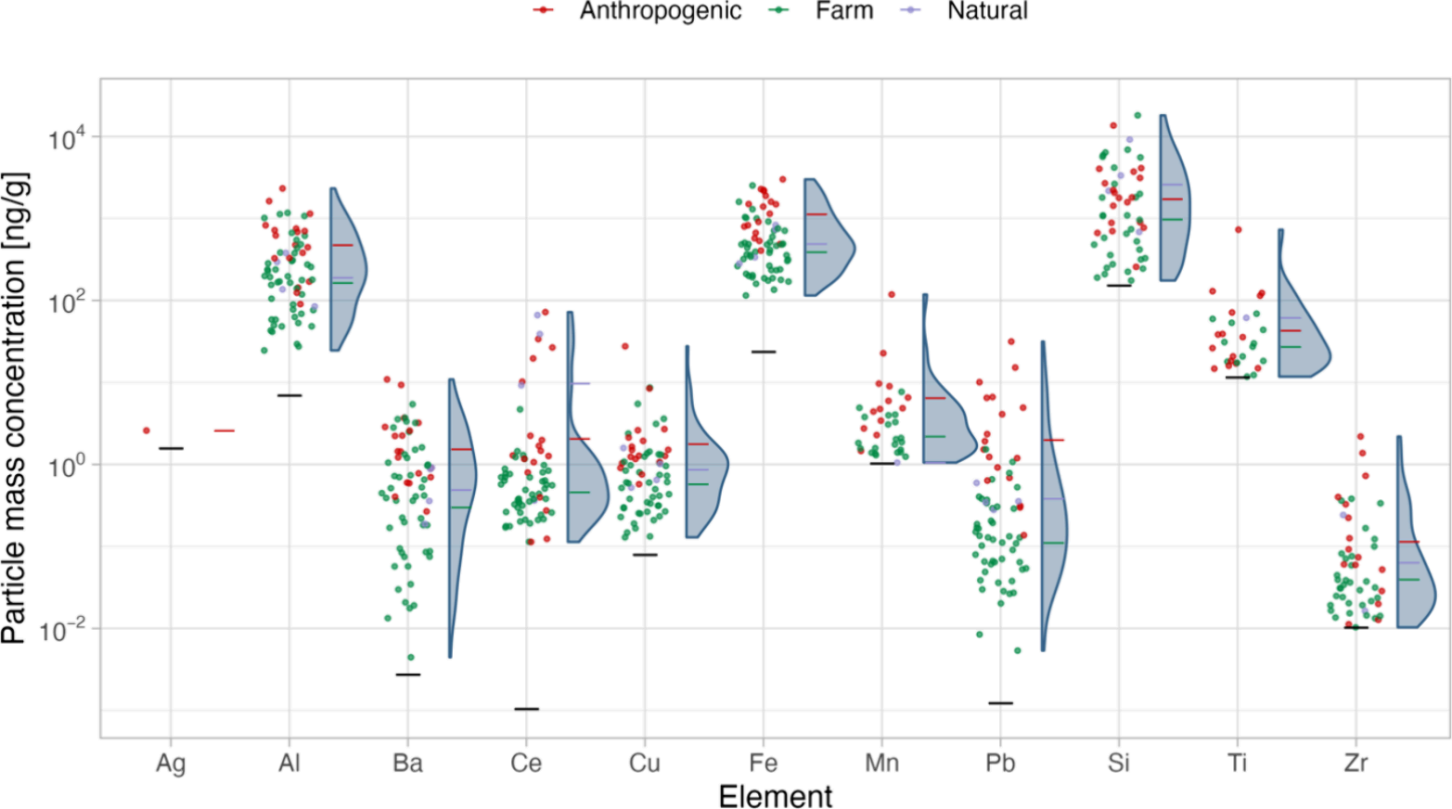
Particle mass concentrations for each element in ng/g of wet weight mussel tissue. Dots represent the mean of three parallels, colored according to the location’s classification (anthropogenic, farm, or natural). The corresponding density plots are shown to the right; mean values for each group are indicated by horizontal lines. Detection limits for each element are denoted by horizontal black lines.

The elements Ag, Al, Ba, Ce, Cu, Fe, Mn, Pb, Si, Ti, and Zr were selected due to the existence of engineered or incidental particles containing these elements. Cr,- Hg-, and Zn-containing particles were screened in four samples (Repparfjorden 1, Fordefjorden, Rana 1, and Bergen Nygårdsbroen). The resulting concentrations were below the detection limit or at similar, low levels, and for this reason, these were excluded from the survey. As shown in Figure 2, concentrations spanned an order of magnitude or more for each element except for silver, where only a few samples (5 of 69) contained particles above the detection limit in terms of particle number concentration. Particle mass concentrations were in the range of a few ng/g up to tens of μg/g depending on the element, with Si, Fe, Al, Ti, and Mn at highest concentrations. Exact values for mean, minimum, and maximum particle mass and number concentrations are presented in Table 1; the corresponding detection limits are shown in Table S4.

**Table 1:** Mean, Minimum, and Maximum of Particle Number and Mass Concentrations per Wet Weight Mussel Tissue Determined for Each Element^a^

	particle number concentration [#/g]			particle mass concentration [ng/g]		
**element**	**mean**	**min–max**	% > *L*_d_	**mean**	**min–max**	% > L_d_
Ag	6.6e6	(3.4e6–1.4e7)	7	2.6		1
Al	7.5e8	(1.5e8–2.8e9)	97	370	(25–2,300)	100
Ba	1.2e7	(2.9e6–4.9e7)	52	1.3	(0.0045–11)	100
Ce	1.2e8	(7.3e6–1.6e9)	100	4.6	(0.11–72)	100
Cu	1.3e7	(3.6e6–1.2e8)	70	1.6	(0.13–28)	99
Fe	7.5e8	(2.4e8–2.1e9)	100	720	(120–3000)	100
Mn	3.0e7	(6.7e6–1.8e8)	46	7.2	(1.1–120)	51
Pb	3.0e7	(2.9e6–2.3e8)	77	1.5	(0.0054–32)	100
Si	7.8e8	(2.8e8–2.2e9)	26	2,500	(180–18,000)	77
Ti	5.5e7	(5.4e6–3.3e8)	100	65	(12–730)	42
Zr	8.2e6	(3.2e6–1.8e7)	23	0.16	(0.010–2.2)	75

a % > *L*_d_ indicates the percentage of locations out of 69 at which the mean value was above the detection limit.

The lack of method intercalibration complicates the direct comparison of NP concentrations between environmental studies. Furthermore, as number concentrations increase with decreasing size whereas the mass of a single particle increases with cubic dependency, upper and lower detection limits are parameters of critical importance. However, Ag, Ce, Cu, and Ti particle concentrations found in this work were in a similar range as reported previously in bivalves.^12,13,15,17,19,21^ Concentrations of Al-, Fe-, and Si-containing NPs in the same range were found in a mussel tissue reference material,^20^ whereas Ba, Mn, and Pb to our knowledge have not been reported for filter feeders.

**Figure 3:**
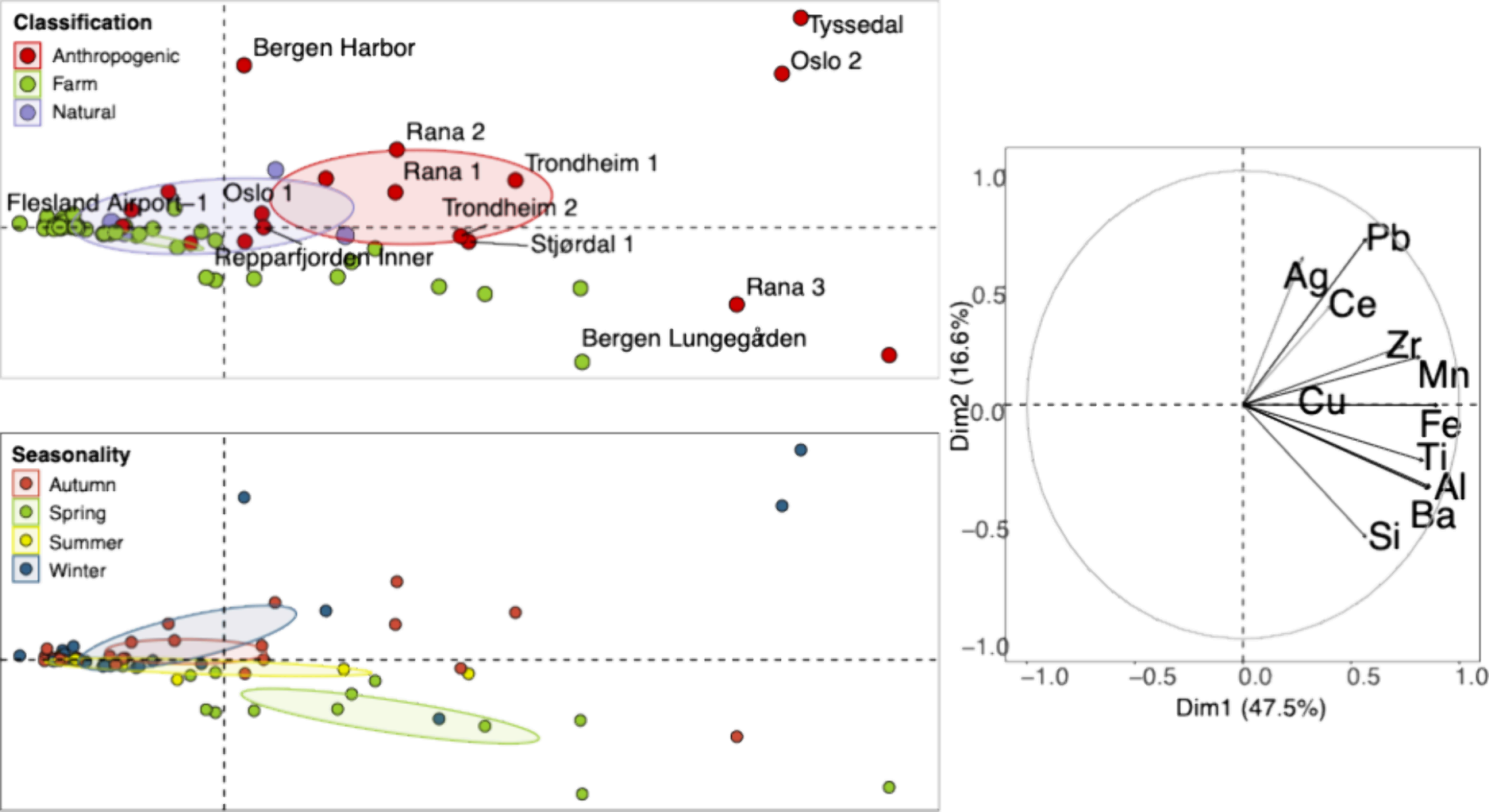
PCA applied to standardized particle number concentrations. To the right is the loading plot showing the relative contributions and correlations between the different elements. To the left are the score plots showing the mean concentration of each sample location with color according to classification (top) or seasonality (bottom). Potentially anthropogenically impacted locations are appended the geographic name. Colored circles indicate 95% confidence ellipses of the mean.

As shown in Figure 2 and Figure S1, respectively, mostly similar ranges of particle mass and number concentrations were observed across the differently classified locations. However, locations potentially impacted anthropogenically trended toward higher concentrations, whereas mussel farms leaned toward lower concentrations. The latter finding might be explained by different growth rates and limited age ranges of farmed mussel and by mussel farms being placed artificially on nets suspended in the water column further from potential sources of particles such as runoff and resuspension. Regulations also result in their placement outside polluted areas. Concentrations of Al-, Ba-, Cu-, Fe-, and Si-containing NPs were largely comparable across the groups, suggesting predominantly natural sources for these elements, whereas Ag, Ce, Mn, Pb, Ti, and Zr trended toward higher concentrations in the anthropogenic group. PCA (principal component analysis) was used to further examine these variabilities, and results are displayed in Figure 3, whereas a heatmap appended hierarchical agglomerative clustering depicts the raw data (Figure S6).

Pb, Ag, and Ce, in order of decreasing importance, were grouped together on the biplot in Figure 3, orthogonal to Al, Ba, Si, and Ti, with Fe, Mn, and Zr falling between. The projection of the sample locations distributes natural locations to the left and parallel with Si, Ba, Al, Ti, and Fe, thus denoting that NPs of these elements are abundant in farms and natural locations (Figure 3). This corroborates with particles previously determined in fjord seawater using SP-ICP-MS.^34^ In contrast, potentially anthropogenically impacted locations are distributed to the right, underlining overall higher concentrations, and parallel to elements of the first group, Ag, Pb, and Ce, suggesting anthropogenic inputs of these elements. A similar grouping structure was observed in the dendrogram in Figure S6. However, the spread of the points illustrates that there are substantial differences between the composition and concentrations at the different anthropogenic sites. Out of these three elements, especially Pb had the highest concentrations in urban and industrially impacted locations (Tyssedal and throughout Ranfjorden: Rana 1–3) (Figures S2 and S3). This was echoed by Pb having the largest PCA loading (Figure 3), being the element most associated with anthropogenic locations, which has been also described for its dissolved counterpart.^35^ Ag was predominantly detected at locations within the urban areas of Bergen and Oslo but was also detected above the detection limit in single parallels in locations anticipated to be free from anthropogenic impact (Figures S2 and S3). Ag NPs in the environment are expected to be primarily of anthropogenic origin,^36^ as supported by the present findings. However, natural sources do also exist.^37^ Silver is used in medicine, textiles, cosmetics, printing,^38^ food contact materials,^39^ and many other applications.^40^ Ce, Zr, Mn, and Ti were found at elevated levels in both natural and anthropogenic locations, consistent with both anthropogenic and natural sources. Ti particles are used in coatings^24^ as well as in consumer products including toothpaste, cosmetics, sunscreen, food, plastics, and more.^41^ Zr is enriched in the particulate fraction in road dust and may, e.g., originate from brake or road wear,^23^ which is a potential source in the locations with elevated levels. It is also present naturally in many minerals and as its oxide.^42^ Ce is among the most abundant rare-earth elements and, in an anthropogenic context, used as a fuel additive, in paints, and for chemical and mechanical planarization.^43^ The industrially impacted location Tyssedal represented an outlier in terms of multiple elements and showed orders of magnitude higher mass and nearly 2-fold larger size per particle for Ti (Figure S4), which may be attributed to the TiO_2_ melting plant in the vicinity. Ba was elevated in Ranfjorden and nearby a construction site in Bergen but also at lower concentrations at several farmed locations and at a sandy beach (Stjo̷rdal). Ba occurs in silicate minerals and is associated with mining tailings^44,45^ but may also precipitate in seawater as its sulfate, BaSO_4_.^46^ The mining waste-impacted Ranfjorden showed increased concentrations of, e.g., Pb, Ba, Ti, Fe, Ce, and Mn. However, similar elevated concentrations were observed in some natural locations. Cu was mostly low across all locations, with the highest levels near a fish farming location, utilizing Cu impregnation due to its antifouling properties.^47^ Although efforts are under way to discriminate specific anthropogenic NPs from natural NPs using SP-ICP-TOF-MS, there is still limited information regarding the composition and abundance of the huge variety of natural and anthropogenic NPs in the environment.^48^ For this reason, distinguishing anthropogenic from natural NPs in unknown environmental samples remains problematic. Analytical electron microscopy, for example, could help elucidate the speciation of NPs; however, this would be impractical for the extensive sample set with mostly low NP concentrations in the current work. Samples from the same geographic location often exhibited similar elemental composition and were grouped together on the dendrogram in Figure S6, demonstrating potential for environmental fingerprinting based on elemental particle composition. Overall, the highest NP concentrations were typically found at anthropogenic locations, although they were not markedly higher than the highest concentrations observed at nonanthropogenic sites. The distinct difference in the size distribution of Ti-containing particles also suggests that NP speciation differs. As there are no limits for NPs in biota as food, a risk assessment for human consumption was not performed.

The study also examined two other factors influencing contaminant levels in mussels in addition to location: sampling time and shell size. This allowed an explorative assessment of seasonal variations and acted as proxies for age in examining bioaccumulation. Seasonality appended to the PCA score plot in Figure 3 revealed that samples collected in the spring and, to a lesser extent, the summer are distributed to the right and parallel to natural elements Si, Ba, Al, and Ti, indicating overall higher concentrations. Figure S7 details the individual relationships. Similarly, concentrations of total metals have been reported to be higher in the spring.^49^ This is influenced by environmental factors such as stratification in the water column resulting from melting and seasonal fluctuations in the total nutrient pool, supporting primary productivity. Additionally, blue mussels are often affected by starvation in winter periods,^50^ during which time the filtration rate is reduced.^51^ Lastly, both spawning season^4,52^ and starvation affect the body burden due to the loss of soft tissue. As a result, seasonal fluctuations can be greater than long-term trends,^4^ and all these natural variations represent noise, which complicates the identification of spatial trends.

To examine the potential for bioaccumulation of particles with mussel age, shell length was recorded as a proxy for age as it not affected by spawning (Figure S8).^53^ While mussels may concentrate NPs from seawater (resulting in higher concentrations of NPs within the mussels compared to the surrounding seawater), negative or no correlations were found between shell length and particle concentrations, indicating little or no bioaccumulation. Thus, the determined NP concentrations may represent a snapshot of the current environmental status. This corroborates with NPs accumulating mostly in the digestive glands, of which most are depurated,^54−56^ and evidence indicating biodilution rather than bioaccumulation for, e.g., Ag and Ti NPs.^11^ A limitation of the current study was a relatively narrow range of sizes (mostly 3–5 cm, with min–max between 2.4 and 7.4 cm), with the mussel length being confounded by both seasonality and location as well as accumulation being dependent on NP speciation. The ingestion rate may also be higher for smaller mussels than for larger mussels,^52^ and e.g., the digestive gland to tissue ratio may be different. Thus, further studies are required with specific sampling designs to control size, location, and seasonality using a broader variety of NPs.

### Total Metals

**Figure 4:**
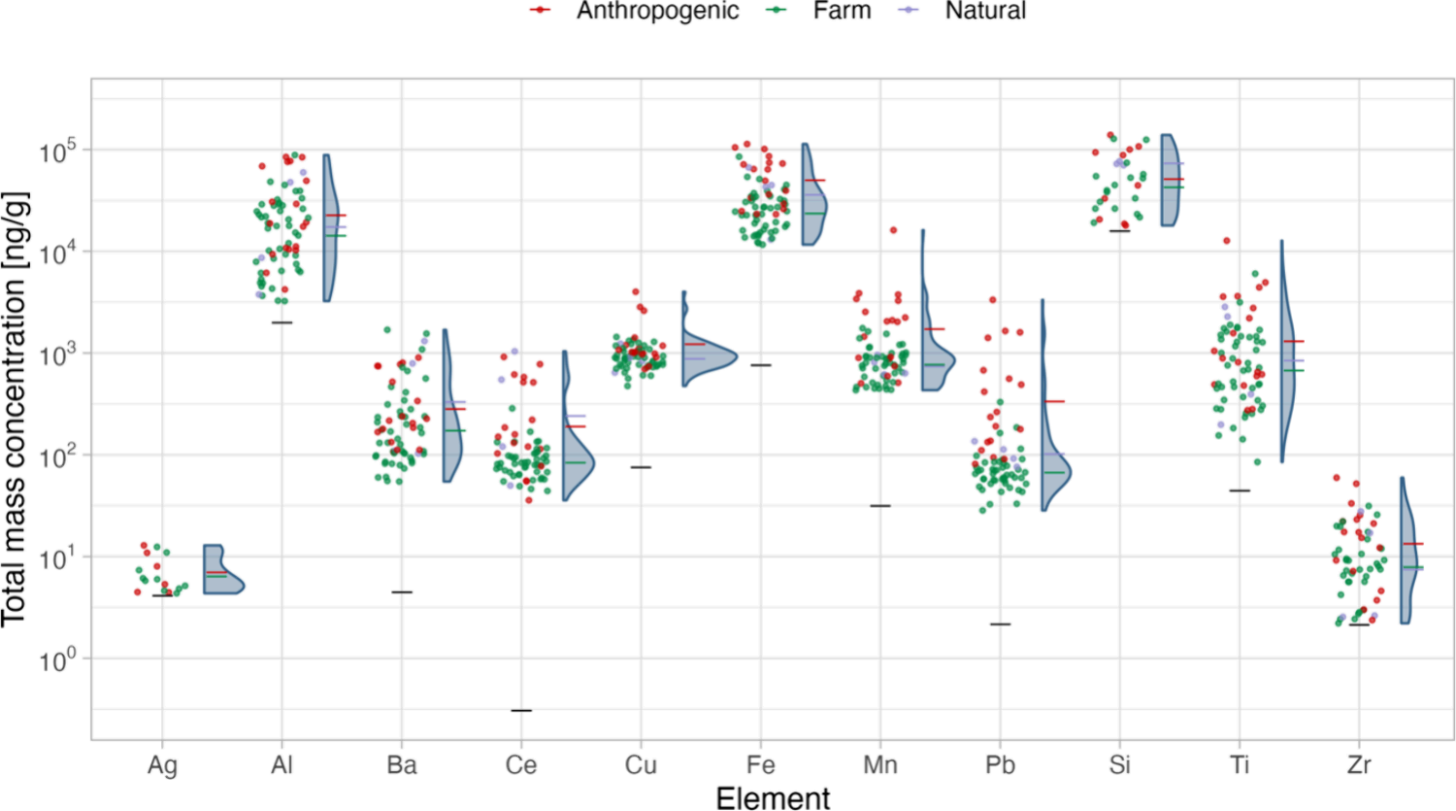
Total metal concentrations for each element in nanograms per gram of wet weight mussel tissue. Dots represent the mean of each sample, colored according to the location’s classification. The corresponding density plots are shown to the right; the mean value across locations is indicated by horizontal lines. The detection limit for each element is denoted by horizontal black lines.

For all elements, total metal concentrations (Figure 4) were orders of magnitude higher than particle mass concentrations (Figure 2), with mean particle fractions mostly around 1% and at most 23% of the total metal concentration, as shown in Table S4. This corroborates with the study by Xu et al. that reported NP mass fractions of 1.25–1.80% for Ag, 1.03–1.38% for Ti, <0.9% for Cu, and <0.5% for Zn in five types of molluscs.^21^ In contrast, one study found Ti-containing NPs to account for 44–55% of the total Ti concentration in marine shellfish.^57^ The authors suggested that the other Ti may be in the form of relatively large particles, dissolved Ti ions, or NPs smaller than the detection limit. Further, the study found that the particle number concentrations of Ti-containing NPs were significantly correlated with Ti concentrations in all tested mussels (*P* < 0.01). Xiao et al. reported a particulate fraction of 13% for Ti and 5% for Ag in various aquatic organisms including mussels and no significant correlation between ionic and particulate forms.^11^ In another study focusing on metal NPs in clams and oysters, NP concentrations accounted for 3.4–50% of the total metal content with the detectable types of NPs being Y, La, Ce, Pr, Gd, and Nd.^19^ It should be noted that the particulate fraction and correlations with dissolved elements are highly dependent on the working range of the method employed and that discrepancies in particle fractions between studies could be linked to methodical differences, also including sample preparation and the use of sonication.

The correlation between total metal and particle mass concentrations differed between elements. For silver, no correlation was found, and for Si, Ba, and Zr, *R*^2^ was below 0.2 (Table S4). This may indicate that particle and dissolved sources may be distinct for these elements. On the other hand, Pb, Mn, and Ti were strongly correlated, suggesting that dissolved and particle sources may co-occur for these elements. A large fraction of NPs below the detection limits or formation of particles (as reported elsewhere for Ti^55^ and Ag^54^) may result in increased correlation with ionic elements. The specificity of SP-ICP-MS for NPs in the presence of ionic concentrations has not been systematically investigated for environmental samples. A hypothesis for lead, in particular, is that the adsorption of its ionic form to other particles can result in it being detected as particulate.^58^ For concentrations, similar trends between total metals and particles are observed for the location classifications, especially Ag, Cu, Pb, and Mn being correlated with anthropogenic sources.

This study represents the first extensive survey of NPs and total metals of 11 distinct elements in blue mussels using (SP-)ICP-MS, demonstrating its usefulness for environmental monitoring. Concentrations varied substantially across different environmental contexts. Ag, Pb, Ce, Cu, and Zr were linked to urban and industrially impacted locations, whereas for other elements (Al, Ba, Fe, Mn, Si, and Ti), natural sources appeared to be more prominent. The industrial location, Tyssedal, represented an outlier with high concentrations of Ti-NPs of substantially larger sizes than all other locations, implying the impact of a local anthropogenic source. Farmed mussels exhibited the lowest NP concentrations. The study further denoted the complexity added by large natural variations and environmental dynamics affecting the concentrations of NPs in the blue mussel samples. Seasonality was linked to substantial variability, whereas overall low concentrations and no correlation with mussel shell length (predominantly in the range of 3 to 5 cm), as a proxy for age, resonate with studies indicating the low bioaccumulation potential of this species for NPs. Thus, concentrations in mussels might represent an environmental snapshot. It should be investigated whether other organisms such as fish are more suitable as bioindicators for NPs in the marine environment as the liver may be a target organ.^59^ As there are currently no established safe limits for NPs within environmental or regulatory frameworks, it is challenging to make definitive statements about their safety concerning food or assess their environmental effects. However, our data suggest that the levels of NPs in mussels and especially those for human consumption are low, although elevated levels are found for specific elements in certain locations.

## Supporting Information Available

Sampling dates, locations, classifications, and mean lengths (Table S1); instrumental parameters (Table S2); detection limits (Table S3); range of particle fractions and correlations with total metals (Table S4); assumed densities and elemental fractions for size calculations; particle number concentrations per element (Figure S1); particle mass concentrations per location (Figure S2); particle number concentration per location (Figure S3); mean size per location (Figure S4); total metal concentrations per location (Figure S5); dendrogram and heatmap of particle number concentrations (Figure S6); particle number concentration vs sampling date (Figure S7); particle mass concentrations vs mean length (Figure S8); and total metal concentrations vs mean length (Figure S9) (PDF)
