# Supplementary material for: Determination of Nanoparticles and Elements in Blue Mussels (Mytilus edulis) along the Norwegian Coastline
Source: J Agric Food Chem. 2024 Nov 7;72(46):25481–9. doi: 10.1021/acs.jafc.4c04721 (PMC11583318; doi:10.1021/acs.jafc.4c04721)
Supplement: Supplementary file 1 — jf4c04721_si_001.pdf [file jf4c04721_si_001.pdf]

Supplementary information: Determination of nanoparticles and elements in blue mussels (*Mytilus edulis*) along the Norwegian coastline

Are Sæle Bruvold[a,b,]*, Stig Valdersnes[a,b], Andre Marcel Bienfait[a], Monica Sanden[a], and Katrin Loeschner [c]

[a] *Institute of Marine Research (IMR), PO Box 1870 Nordnes, N-5817 Bergen, Norway*

b University of Bergen, Department of Chemistry, PO Box 7803, N-5020 Bergen, Norway,

c Technical University of Denmark, National Food Institute, Kemitorvet 201, DK-2800 Kgs Lyngby, Denmark

* email: arebru@gmail.com

Supplementary Table 1 Locations, position, classification, mean length and sampling date for each pooled mussel sample.

Location	Latitude	Longitude	Classification	Mean length	Sampling date
Lillesand 1	58.2094	8.2924	Farm	5.8	23.11.2020
Forsand 1	58.9432	6.1220	Farm	3.9	30.11.2020
Oslo 1	59.9078	10.6422	Anthropogenic	5.5	28.2.2021
Oslo 2	59.9199	10.6723	Anthropogenic	5.1	28.2.2021
Austevoll 1	60.0882	5.2627	Natural	3.9	24.9.2020
Tyssedal	60.1197	6.5563	Anthropogenic	4.0	22.12.2020
Bergen Aquaculture	60.2397	5.2542	Anthropogenic	2.4	30.10.2020
Flesland Airport-1	60.2979	5.2081	Anthropogenic	5.8	29.9.22
Flesland Airport-2	60.2986	5.2077	Anthropogenic	6.1	29.9.22
Bergen Nygårdsbroen	60.3804	5.3342	Anthropogenic	4.7	8.5.2021
Bergen Lungegården	60.3846	5.3404	Anthropogenic	4.4	8.5.2021
Bergen Harbor	60.3990	5.3113	Anthropogenic	4.6	1.2.2021
Dale 2	61.3515	5.3492	Farm	6.4	3.5.2021
Dale 3	61.3515	5.3492	Farm	6.3	29.6.2020
Dale 1	61.3749	5.4176	Farm	6.3	29.6.2020
Førdefjorden 2	61.4760	5.4155	Natural	7.4	31.8.2017
Førdefjorden 1	61.4881	5.4403	Natural	6.1	31.8.2017
Trondheim Harbor	63.4395	10.3997	Anthropogenic	3.1	13.9.2021
Trondheim 2	63.4479	10.4232	Anthropogenic	4.1	13.9.2021
Trondheim 1	63.4486	10.4271	Anthropogenic	4.6	13.9.2021
Stjørdal 1	63.4703	10.8814	Anthropogenic	5.6	12.7.2021
Rissa 1	63.5806	9.9488	Farm	6.4	3.5.2021
Mosvik 1	63.8476	10.7387	Farm	5.0	7.12.2020
Lysøysundet 1	63.8906	9.9536	Farm	5.8	8.6.2020
Åfjord 2	63.9424	10.1838	Farm	5.2	2.2.2021
Åfjord 3	63.9424	10.1838	Farm	5.3	25.5.2021
Åfjord 4	63.9424	10.1838	Farm	5.7	30.11.2020
Åfjord 1	63.9530	10.0043	Farm	4.7	19.4.2021
Nord 3	64.4595	11.1968	Farm	5.5	7.12.2020
Nord 1	64.4896	11.2546	Farm	5.7	7.9.2020
Nord 2	64.4896	11.2546	Farm	5.7	7.12.2020
QC (Namsos 1)	64.6199	11.3890	Farm	6.0	7.12.2020
Terråk 15	65.0340	12.1859	Farm	5.4	25.1.2021
Terråk 16	65.0340	12.1859	Farm	5.6	6.4.2021
Terråk 17	65.0340	12.1859	Farm	5.3	25.5.2021
Terråk 18	65.0340	12.1859	Farm	4.7	8.9.2020
Terråk 19	65.0340	12.1859	Farm	5.0	2.11.2020
Terråk 12	65.0459	12.1569	Farm	5.6	25.1.2021
Terråk 13	65.0459	12.1569	Farm	5.3	26.4.2021
Terråk 14	65.0459	12.1569	Farm	5.9	2.11.2020
Terråk 10	65.0465	12.1279	Farm	5.4	25.1.2021
Terråk 11	65.0465	12.1279	Farm	4.7	7.9.2020
Terråk 5	65.0467	12.1407	Farm	5.5	25.1.2021
Terråk 6	65.0467	12.1407	Farm	5.7	6.4.2021

Terråk 7	65.0467	12.1407	Farm	5.5	25.5.2021
Terråk 8	65.0467	12.1407	Farm	5.0	8.9.2020
Terråk 9	65.0467	12.1407	Farm	6.1	2.11.2020
Bindalseidet 3	65.0620	12.1000	Farm	5.8	25.1.2021
Bindalseidet 4	65.0620	12.1000	Farm	5.0	7.9.2020
Terråk 3	65.0786	12.4411	Farm	5.7	25.1.2021
Terråk 4	65.0786	12.4411	Farm	5.2	7.9.2020
Bindalseidet 1	65.1131	12.2513	Farm	5.9	25.1.2021
Bindalseidet 2	65.1131	12.2513	Farm	5.0	7.9.2020
Terråk 1	65.1841	12.3922	Farm	5.3	25.1.2021
Terråk 2	65.1841	12.3922	Farm	5.0	7.9.2020
Visthus 1	65.6624	12.6403	Farm	6.4	6.12.2020
Vevelstad 1	65.6653	12.6500	Farm	3.9	7.12.2020
Tro 1	65.8157	12.5823	Farm	5.9	6.12.2020
Leirfjord 1	66.0506	12.9552	Farm	5.5	25.1.2021
Leirfjord 2	66.0506	12.9552	Farm	5.5	7.12.2020
Rana 3	66.2802	14.0347	Anthropogenic	4.6	1.11.2021
Rana 2	66.3118	14.1286	Anthropogenic	5.4	1.11.2021
Rana 1	66.3162	14.1232	Anthropogenic	5.2	1.11.2021
Hamnnes 1	69.7922	20.6022	Farm	5.1	23.2.2021
Hamnnes 2	69.7922	20.6022	Farm	5.0	18.5.2021
Hamnnes 3	69.7922	20.6022	Farm	5.3	7.9.2020
Repparfjorden Inner	70.4571	24.2775	Anthropogenic	4.6	19.9.2017
Repparfjorden Outer	70.5206	24.1907	Anthropogenic	4.9	19.9.2017
Repparfjorden Control	70.6354	24.6918	Natural	3.4	18.9.2017

Supplementary Table 2: Instrumental parameters for SP-ICP-MS and total metal analysis using an Agilent 8900 instrument. A peristaltic pump speed of 0.1 RPS was used, corresponding to a sample flow rate of approximately 0.35 mL/min.

**Element**	**Ag**	**Al**	**Au** (calibration)	**Ba**	**Ce**	**Cu**	**Fe**	**Mn**	**Pb**	**Si**	**Ti**	**Zr**
**Scan type**	**MS/MS**	MS/MS	MS/MS	MS/MS	MS/MS	MS/MS	MS/MS	MS/MS	MS/MS	MS/MS	MS/MS	MS/MS
**Isotope monitored [m/z]**	**109/109**	27/27	197/197	137/137	140/140	63/63	56/56	55/55	208/208	28/28	48/64	90/90
**Reaction gas flow [mL/min]**	**0**	0	0	0	0	0	5.0 H_2	0	0	3.0 H_2	7.0 H_2, 0.10% O_2	0
**RF power [V]**	**1600**	1600	1600	1600	1600	1600	1600	1600	1600	1600	1600	1600
**Sampling depth [mm]**	**8**	8	8	8	8	8	8	8	8	8	8	8
**Octopole bias [V]**	**-8**	-8	-8	-8	-8	-8	-18	-8	-8	-18	-6	-8
**Axial acceleration [V]**	**0**	0	0	0	0	0	0	0	0	0	1	0
**Energy discrimination [V]**	**5**	5	5	5	5	5	0	5	5	0	-15	5

Supplementary Table 3: Detection limits for each element for both single particle and total metal analysis. Internal standard of Rh was used for total metal analysis.

	Single particle			Total metals
**Element**	**Particle mass [ag]**	**Mass concentration [ng/g]**	**Number concentration [#/g]**	**Mass concentration [ng/g]**
Ag	12	1.6	3.3 × 10^6	4.1
Al	116	6.9	1.1 × 10^8	2,000
Ba	127	0.0027	2.8 × 10^6	4.5
Ce	23	0.0010	2.7 × 10^6	0.31
Cu	203	0.079	3.5 × 10^6	75
Fe	417	24	5.4 × 10^7	750
Mn	581	1.0	6.0 × 10^6	32
Pb	89	0.0012	2.7 × 10^6	2.1
Si	2,241	150	2.5 × 10^8	16,000
Ti	52	12	3.8 × 10^6	44
Zr	8	0.010	3.1 × 10^6	2.1

Supplementary Table 4: Mean, minimum and maximum particle mass fraction in percent and correlation between particulate and total mass concentrations across all samples for each element.

	Particle fraction [%]			Correlation
**Element**	**Mean**	**Minimum**	**Maximum**	**R-squared**
Ag	0.0	0.0	0.0	NA
Al	1.7	0.0	7.3	0.5
Ba	0.4	0.0	2.7	0.2
Ce	1.3	0.1	12.1	0.5
Cu	0.1	0.0	0.9	0.6
Fe	1.9	0.4	6.3	0.6
Mn	0.1	0.0	1.0	0.9
Pb	0.4	0.0	2.3	0.9
Si	3.8	0.0	22.5	0.4
Ti	1.1	0.0	7.9	0.8
Zr	1.0	0.0	9.9	0.2

**For silver no samples were above the detection limit both for total metals and particle mass concentration.*

Supplementary Table 5: Densities and elemental fractions used to calculate mass equivalent spherical diameters.

Element	Density	Element fraction
Au	19,32	1,00
Al	2,56	0,10
Mn	4,25	0,63
Pb	6,29	0,68
Fe	4,3	0,63
Si	2,65	0,47
Ti	4,17	0,60
Ce	7,22	0,81
Zr	5,68	0,74
Cu	6,31	0,80
Ba	2	1,00

Ag	10,49	1,00

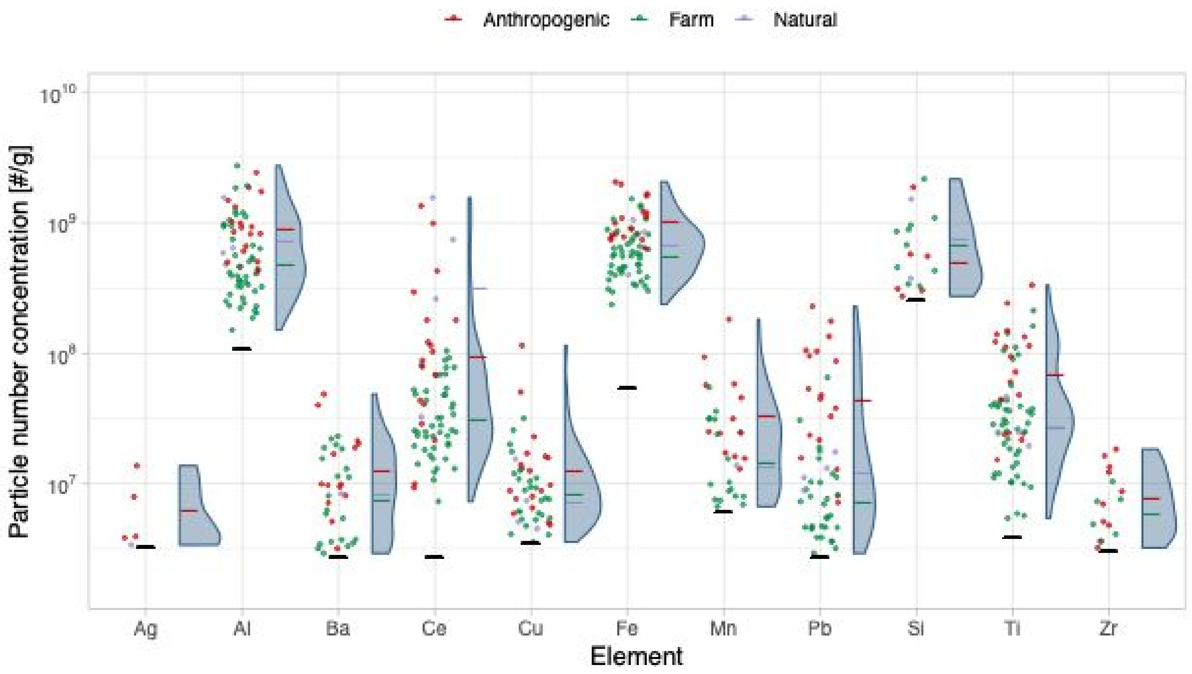

Supplementary Figure 1: Particle number concentrations for each element per wet weight mussel tissue. Dots represent the mean of three parallels, colored according to the locations' classification. The corresponding density plots are shown to the right, mean values across all samples are indicated by horizontal lines. Detection limits for each element are denoted by the horizontal black lines.

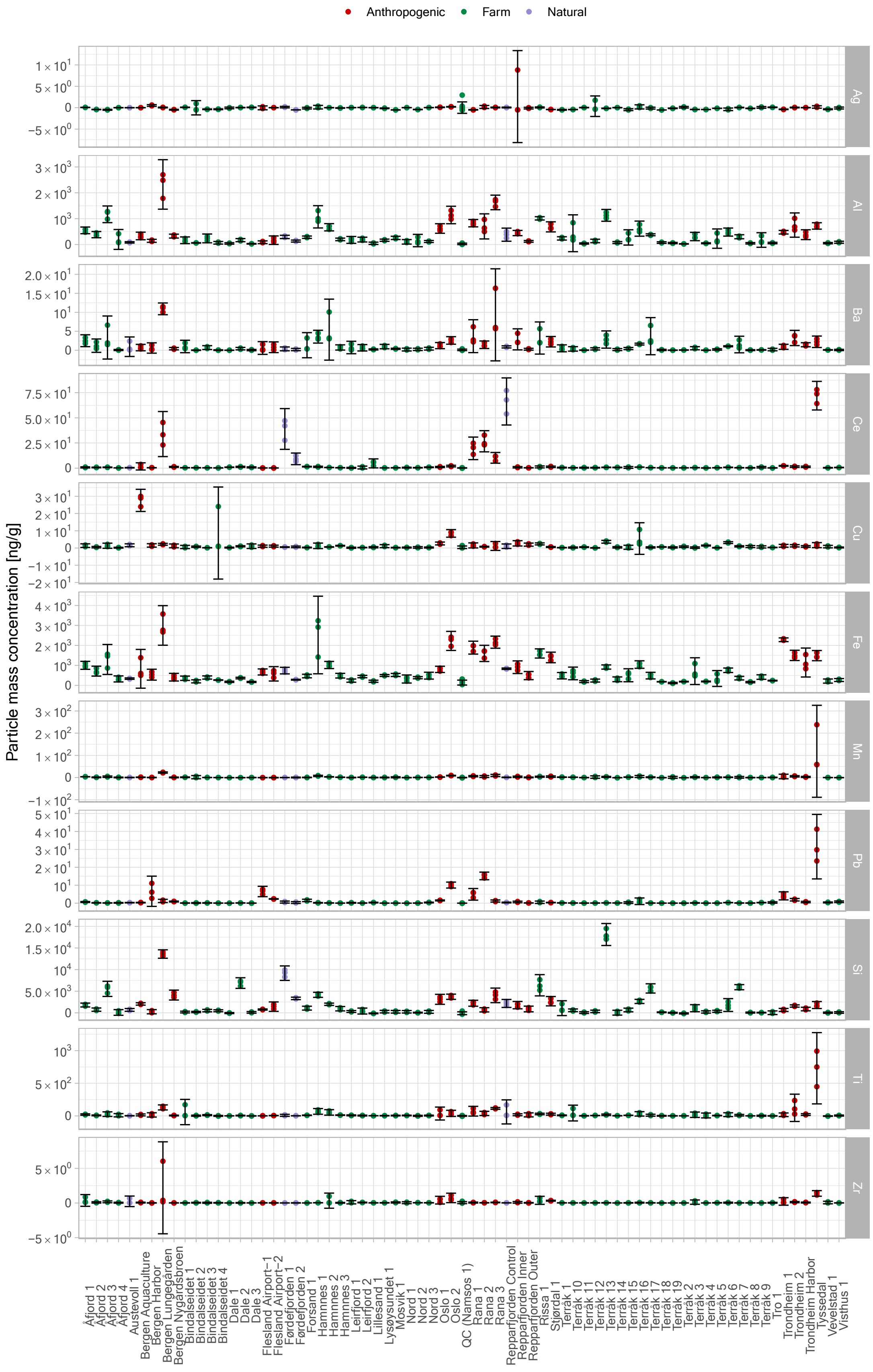

Supplementary Figure 2: Particle mass concentrations for each location. Each data point representing a replicate. The error bars represent two times the standard deviation.

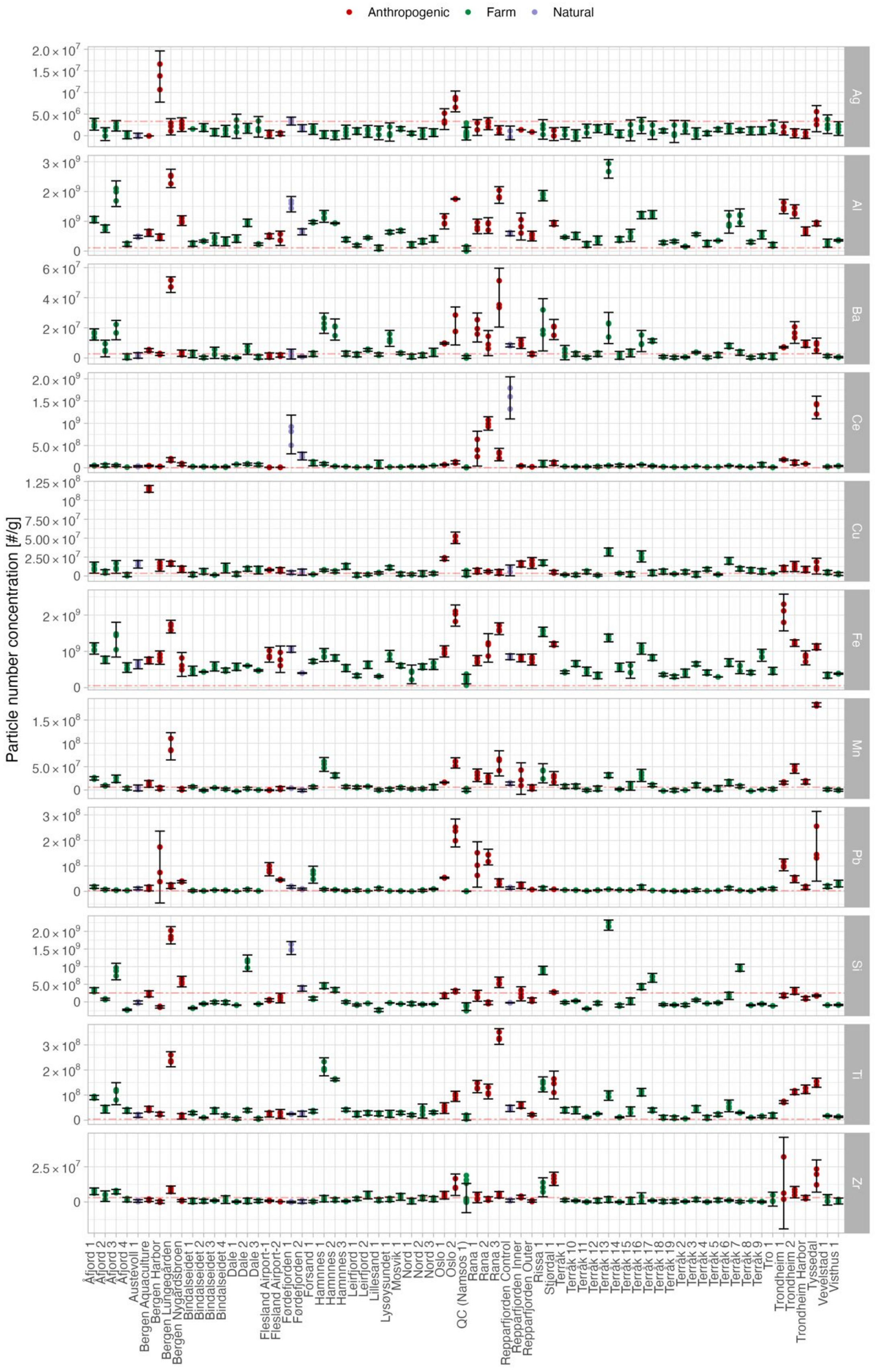

Supplementary Figure 3: Particle number concentrations for each location, error bars representing two times the standard deviation.

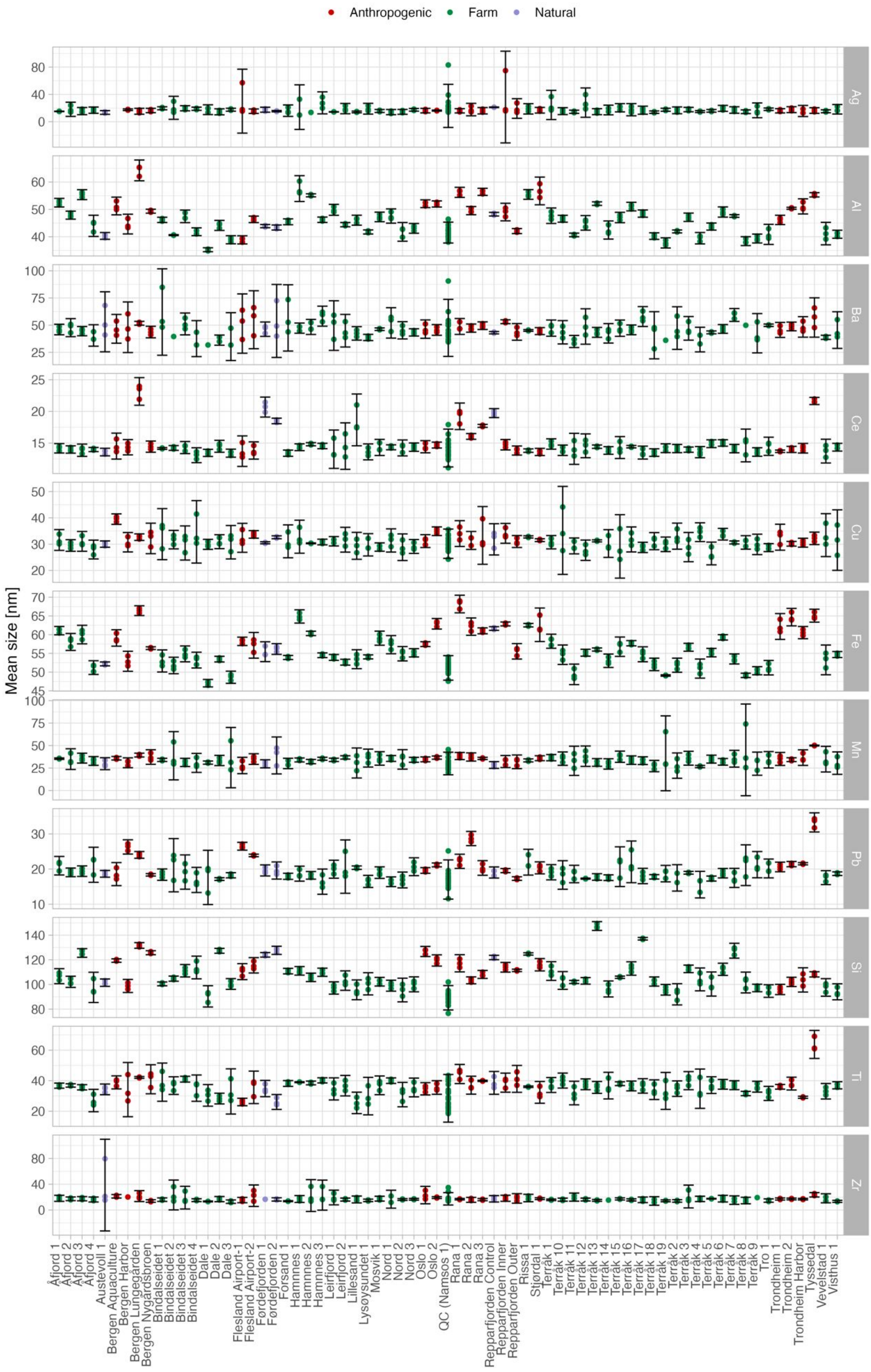

Supplementary Figure 4: Mean size as mass-equivalent spherical particle diameter in nanometers for each location, assumptions about composition shown in Supplementary Table 5.

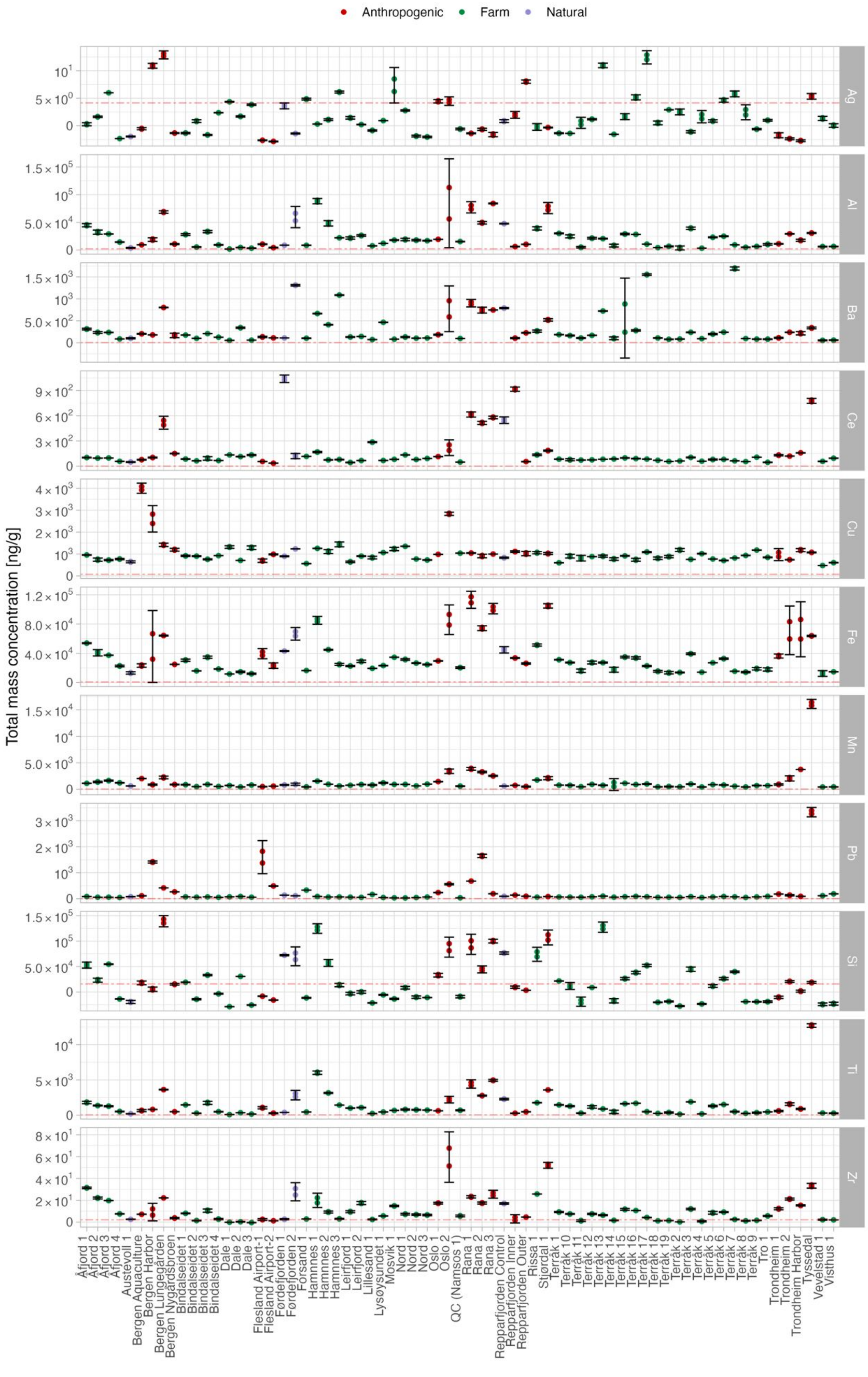

Supplementary Figure 5: Total metal concentrations for each location.

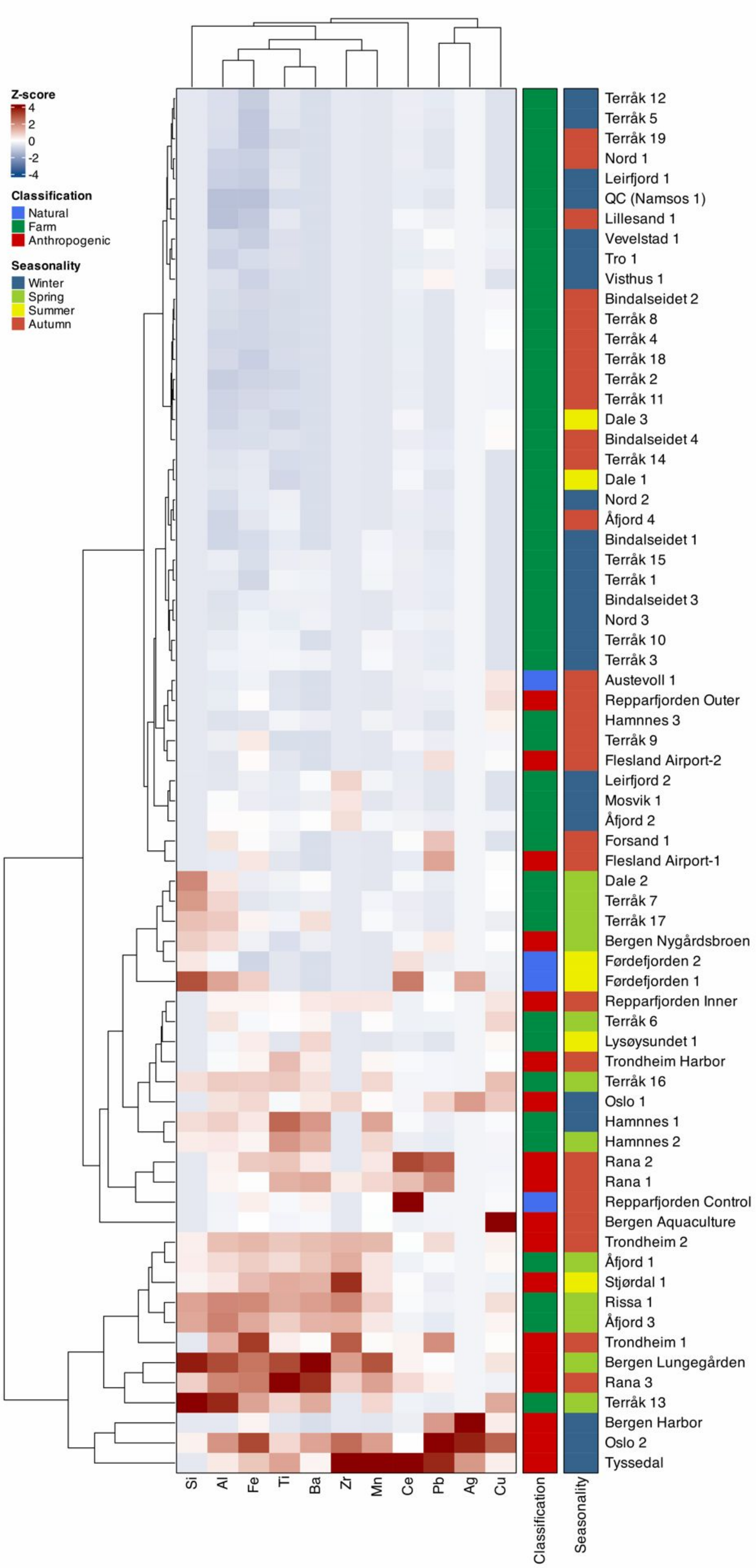

Supplementary Figure 6: Agglomerative hierarchical clustering applied with heatmap indicating the particle number concentration of each element, the row dendrogram indicates clusters across locations, whereas columns indicate clustering among the elements.

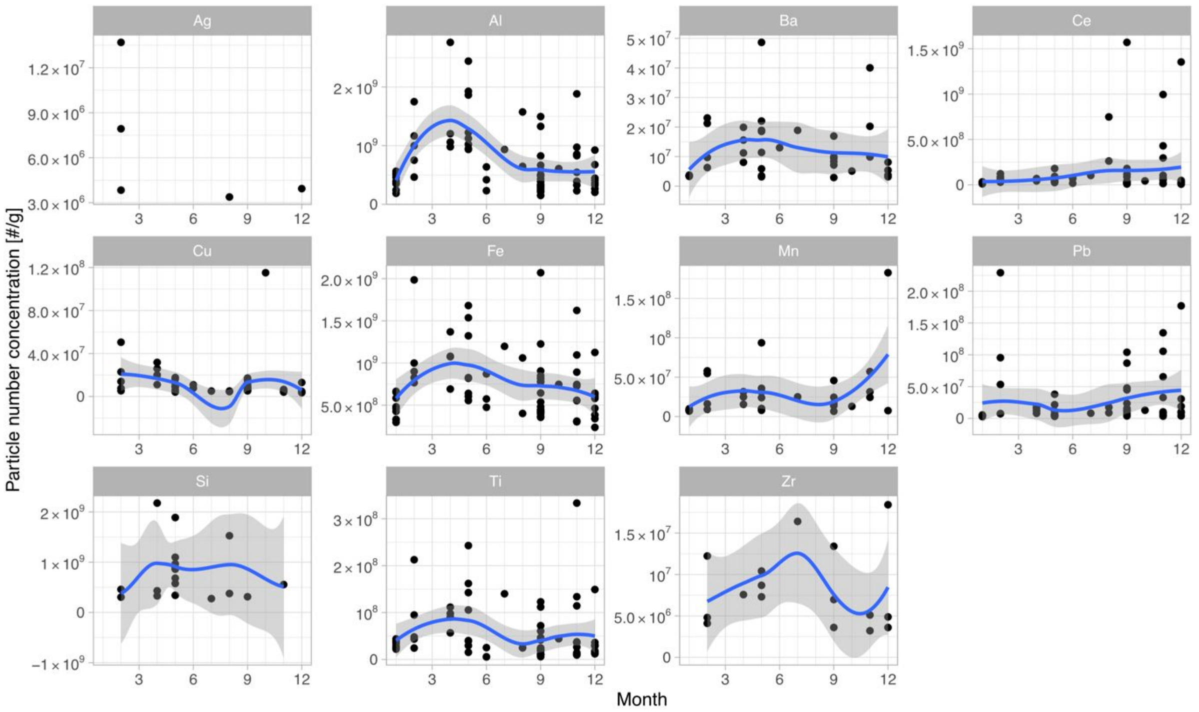

Supplementary Figure 7: Particle number concentrations for each element plotted versus the month at which sampling took place.

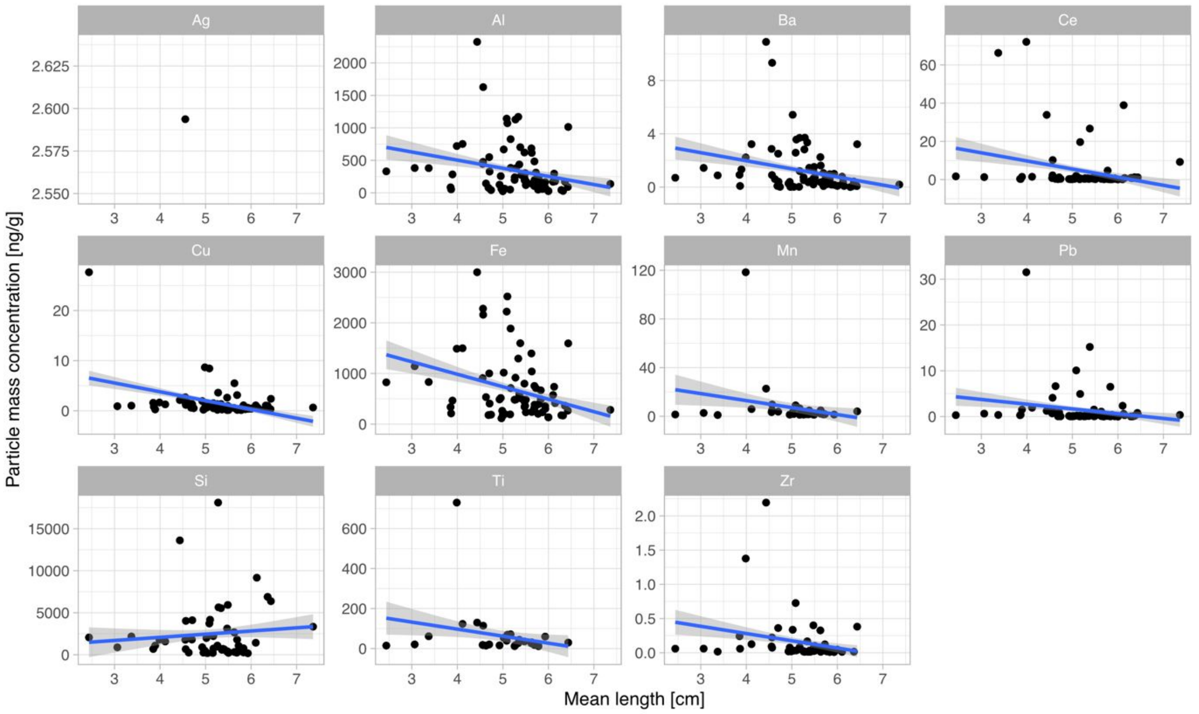

Supplementary Figure 8: Particle mass concentrations plotted against the mean length of the mussels for each location. The blue line indicates the ordinary least squares linear fit, shaded area indicating 95% confidence level interval for predictions.

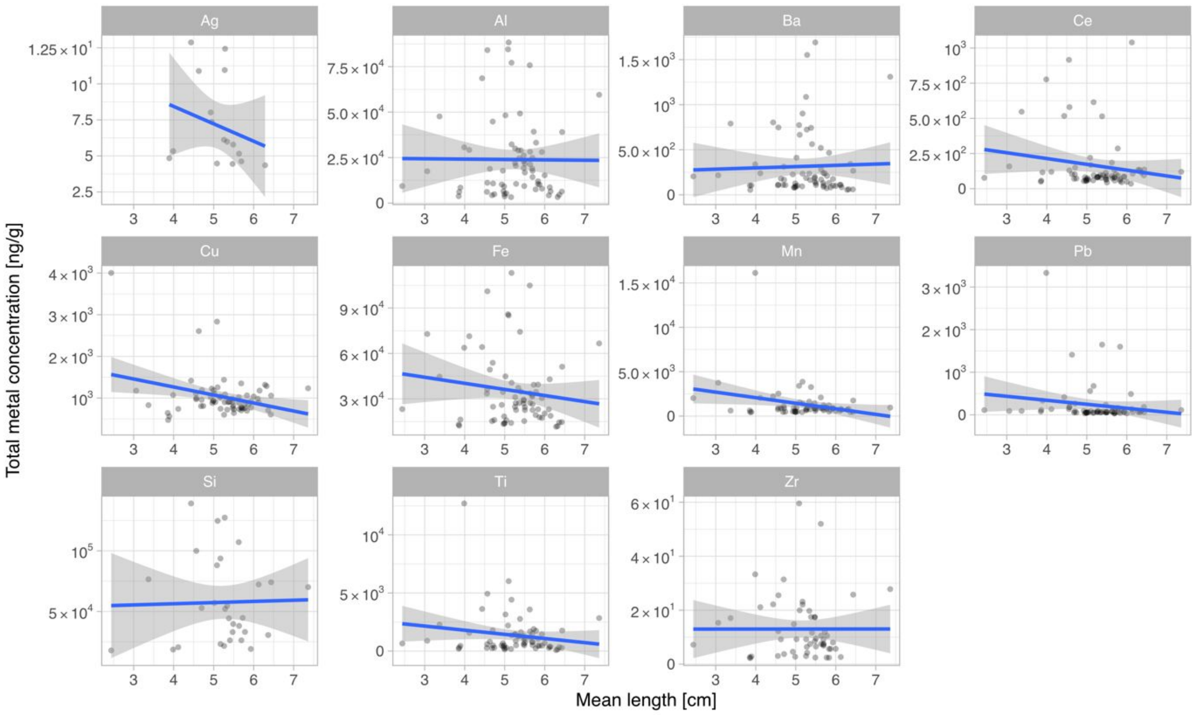

Supplementary Figure 9: Total metal concentrations plotted against the mean length of the mussels for each location.
